# Further development of the 12-item EDE-QS: identifying a cut-off for screening purposes

**DOI:** 10.1186/s12888-020-02565-5

**Published:** 2020-04-03

**Authors:** Katarina Prnjak, Deborah Mitchison, Scott Griffiths, Jonathan Mond, Nicole Gideon, Lucy Serpell, Phillipa Hay

**Affiliations:** 1 School of Medicine, Western Sydney University, Sydney, Australia; 2 Centre for Emotional Health, Department of Psychology, Macquarie University, Sydney, Australia; 3 Physical Appearance Research Team, Melbourne School of Psychological Sciences, University of Melbourne, Melbourne, Australia; 4 Centre for Rural Health, University of Tasmania, Launceston, TAS Australia; 5 Suffolk Family Focus Psychology Service, Norfolk and Suffolk NHS Foundation Trust, Suffolk, UK; 6 Research Department of Clinical, Educational and Health Psychology, University College London, London, UK; 7 North East London NHS Foundation Trust, Essex, UK; 8 Camden and Campbelltown Hospitals, SWSLHD, Campbelltown, Australia

**Keywords:** EDE-QS, Eating disorders, Screening, ROC analysis, Discriminant validity

## Abstract

**Background:**

The Eating Disorder Examination – Questionnaire Short (EDE-QS) was developed as a 12-item version of the Eating Disorder Examination Questionnaire (EDE-Q) with a 4-point response scale that assesses eating disorder (ED) symptoms over the preceding 7 days. It has demonstrated good psychometric properties at initial testing. The purpose of this brief report is to determine a threshold score that could be used in screening for probable ED cases in community settings.

**Methods:**

Data collected from Gideon et al. (2016) were re-analyzed. In their study, 559 participants (80.86% female; 9.66% self-reported ED diagnosis) completed the EDE-Q, EDE-QS, SCOFF, and Clinical Impairment Assessment (CIA). Discriminatory power was compared between ED instruments using receiver operating characteristic (ROC) curve analyses.

**Results:**

A score of 15 emerged as the threshold that ensured the best trade-off between sensitivity (.83) and specificity (.85), and good positive predictive value (.37) for the EDE-QS, with discriminatory power comparable to other ED instruments.

**Conclusion:**

The EDE-QS appears to be an instrument with good discriminatory power that could be used for ED screening purposes.

## Background

The Eating Disorder Examination Questionnaire (EDE-Q [1];), derived from the Eating Disorder Examination (EDE [2];), is one of the most widely used and extensively validated self-report instruments for eating disorder (ED) assessment. However, the lengthy administration time of the EDE-Q and the 28-day period over which it captures symptoms may be problematic when monitoring session-by-session outcomes in the context of clinical practice and treatment. Consequently, researchers have developed modified versions of this tool [3]. One such version is the 12-item EDE-QS, which was recently developed employing Rasch’s analysis among individuals with EDs receiving specialist treatment [4]. The EDE-QS removes the open-ended responses to behavioural items and also narrows the reference timeframe from the past 28-days to the past 7-days. Alongside its brevity, this timeframe was adopted to improve recall potential, and positions the EDE-QS as a routine outcome-monitoring instrument for individuals in treatment to aid clinical decision-making via improved and regular feedback [5].

The EDE-QS has demonstrated good internal consistency, test-retest reliability, convergent validity and sensitivity in a mixed sample of individuals with probable ED and individuals probably not having an ED, recruited from a university and a charity [4]. Its brevity and psychometric properties provide preliminary support for the use of the EDE-QS as a screening tool for people with EDs in community settings. However, a threshold value was not established for the EDE-QS questionnaire, as it was not originally developed as a screening instrument. Cut-off scores are important for facilitating the detection of individuals likely to be experiencing threshold EDs and differentiating these individuals from those who are unlikely to have ED symptoms. Existing instruments (such as the original EDE-Q) are either too long to be practical to use for screening purposes, do not capture each disorder across the ED spectrum (for instance, SCOFF does not assess BED symptoms), or have inadequate psychometric properties in some populations (e.g. SCOFF in overweight women) [6]. Therefore, the aim of this brief report was to establish and evaluate a cut-off point on the EDE-QS that could be utilized by researchers and clinicians when identifying probable ED cases in community settings.

## Method

### Participants and procedures

A total of 559 people, who were recruited from a university and through a charity that offers support for eating disorders, participated in the EDE-QS validation part of the study first reported by Gideon et al. [4]. The same data are used for the secondary analysis reported in the current study. The majority of participants were women (80.86%) aged between 18 and 34 years (92.31%). Seventy-eight percent of the participants identified as White and 88.90% had tertiary (post-secondary school) education. Fifty-four (9.66%) participants self-reported currently having an ED diagnosis (i.e. they responded with *Yes* to the question “Do you currently suffer from an eating disorder (anorexia nervosa, bulimia nervosa, binge eating disorder, eating disorder not otherwise specified)?”). Of these, 7 (13%) met criteria for restrictive anorexia nervosa (AN-R); two (4%) for binge eating/purging AN subtype (AN-BP); eight (15%) for bulimia nervosa (BN); 10 (19%) for binge eating disorder (BED); and 27 (50%) for other specified feeding and eating disorders (OSFED) based on their responses on the EDE-Q and their Body Mass Index (BMI). Mean BMI in the total sample was 22.35 (*SD* = 4.61), with 12% of participants classified as underweight, 72% as average-weight, 12% as overweight, and 3% as obese. Underweight individuals were not excluded from the subsample of participants who self-reported currently not having an ED diagnosis. Detailed information about recruitment processes and data collection have been presented elsewhere [4]. In brief, an invitation to participate was distributed to a large university in London, UK, and the study was advertised via the Beat - *Beating Eating Disorders* website, a UK’s eating disorder charity that offers support to people with current or former ED difficulties and their families. Participants provided informed consent and completed an online survey.

### Measures

#### Eating disorder examination-questionnaire short

The EDE-QS was developed by Gideon et al. [4] as a 12-item version of the EDE-Q (see below) with a response scale ranging from 0 to 3, that captures essential symptoms of AN, BN and BED. The response scale was shortened during the development of the EDE-QS to reduce the cognitive demand and because respondents were not making full use of the 0–6 scale (some original categories were not used consistently with respondents’ ED severity), as observed in Rasch analysis results [see 4]. Scores of items are summed, ranging from 0 to 36 and higher scores indicate greater ED symptoms. ED symptoms are reported for the preceding seven days. Cronbach’s alpha obtained in this sample was .91 [4] indicating excellent internal consistency.

#### Ede-q

The most recent version of the full EDE-Q [7] is a 28-item measure of ED symptoms and behaviors. Scores on each of four subscales (Restraint, Eating Concerns, Shape Concerns, Weight Concerns) and a Global score may be derived from items assessing core attitudinal features. Participants provide their answers on a scale from 0 to 6, with higher scores indicating greater frequency and/or severity of ED psychopathology over the previous 28 days. The EDE-Q has been validated in various clinical and non-clinical samples [8]. Previous studies showed a cut-off score for “probable” ED amongst young women of 2.3 (in conjunction with the occurrence of binge eating and/or excessive exercise) [9], and a clinical cut-off of a global EDE-Q score ≥ 2.8 [6]. In the current study sample, Cronbach’s alpha was .96 for the global score and .84, .86, .93 and .88 for the Restraint, Eating Concern, Shape Concern, and Weight Concern subscales, respectively.

#### Scoff

The SCOFF [10] is a 5-item measure used to screen for EDs in primary care. Items tap into key symptoms of AN and BN with a dichotomous (Yes/No) response scale. The number of “Yes” responses are summed to create a total score, with a score ≥ 2 indicative of an ED. The SCOFF has been found to have good psychometric properties in international community samples [11]. Cronbach’s alpha in the current sample was .64 [4].

#### Clinical impairment assessment

The CIA (CIA 3.0) [12] was designed to measure psychosocial impairment associated with key ED features in the past 28 days. Sixteen items are answered on a 4-point Likert-type scale summed to compute the global score, with higher scores indicating greater perceived impairment. The CIA has previously been used for ED instrument validation [13, 14] since clinical impairment has shown to be higher among ED clinical samples relative to healthy controls [15], thus supporting the instrument’s criterion validity [16]. The CIA has robust psychometric properties [12]. In the present sample, Cronbach’s alpha was .96.

### Data analysis

The R package “epiR” was used to obtain sensitivity (the proportion of true cases correctly identified by the test), specificity (the proportion of true non-cases correctly identified by the test), positive predictive values (PPV; the proportion of individuals with positive test results who have an ED), and negative predictive values (NPV; the proportion of individuals with negative test results who do not have an ED). The PPVs and NPVs depend upon the prevalence of the disorder (e.g. if the prevalence is < 10% then PPV can be < 0.5) thus there is no defined criterion for classifying PPV or NPV as “acceptable,” or “good” [17]. The package “pROC” was used to compute the Area Under the Curve (AUC; the surface area under the curve which describes the relationship between sensitivity and specificity) statistic and confidence intervals (CI). AUC can obtain values from 0 to 1, with AUC of 0.50 classified as non-informative; between 0.50 and 0.70 as less accurate; between 0.70 and 0.90 as moderately accurate; between 0.90 and 1 as highly accurate; and AUC = 1 is considered as perfect [18]. Two participants (0.36%) with missing values in the question about current ED diagnosis were excluded from the analyses (their Global EDE-Q scores were in a 30th and 38th percentile, indicating their removal would not markedly impact analyses as they did not have extreme results in this variable). Complete data were available for all other variables. Summation scores are more sensitive to missing data than other scoring methods [19], but as there were no missing data for the EDE-QS, summation was considered to be appropriate for this study. Global cut-off scores calculated as the average of item scores are also derived and presented in Table 1 in case of a necessary use by future researchers or clinicians, although for the simplicity in the following text we limit our discussion to global summation scores.

**Table 1:** Mean (SD) of clinical impairment, EDE-Q scores, and eating disorder behaviour for self-reported eating disorder cases (*n* = 54) and non-cases (*n* = 503) in the current study and normative data reported by Mond et al. (2004)

	*Current study*			*Normative data*	
	*Self-reported ED cases (n=54)*	*Self-reported ED non-cases (n=503)*		*ED cases*	*ED non-cases*
	*Mean (SD)*	*Mean (SD)*	*t*	*Mean (SD)*	*Mean (SD)*
Clinical impairment	28.67(12.44)	8.24(8.47)	12.15**		
Global EDE-Q	3.99(1.39)	1.67(1.32)	12.25**	3.09(0.83)	1.30(0.96)
Restraint	3.67(1.69)	1.52(1.46)	9.47**	2.65(1.48)	1.19(1.21)
Eating concerns	3.40(1.54)	0.97(1.22)	11.26**	2.02(0.95)	0.49(0.74)
Weight concerns	4.23(1.43)	1.86(1.59)	12.04**	3.68(1.08)	1.49(1.20)
Shape concerns	4.64(1.44)	2.31(1.63)	11.90**	4.01(0.98)	2.03(1.38)
OBE	44.4%	12.1%	4.63**	25.0%	2.2%
SBE	5.5%	9.7%	-1.23	25.0%	6.0%
Excessive exercising^a^	27.8%	6.4%	3.43*	58.3%	8.2%

*Note.* ED – eating disorders; OBE – objective binge eating (≥ 4 episodes in the last 28 days); SBE – subjective binge eating (≥ 4 episodes in the last 28 days)

^a^ ≥ 20 times in the last 28 days

* *p* < .01

** *p* < .001

Using the self-reported ED diagnosis variable, the number of true positive, false positive, true negative, and false negative cases were determined separately for every possible cut-off score on the EDE-QS, and for the previously established range of cut-off scores on the EDE-Q (from 1.3 to 2.9) [20] and all possible SCOFF scores (from 0 to 5) [21]. Sensitivity and specificity rates were calculated for each of the possible cut-off score in these instruments, and PPV and NPV were also obtained. For the purpose of the current study, only findings for cut-off scores that yielded the highest levels of discriminatory parameters are reported, as the aim was to detect a cut-off score with high sensitivity, specificity and PPV. In addition to this, discriminatory parameters were also derived among female participants solely (*n* = 452).

## Results

### Comparison of ED symptom levels between study subgroups

In the absence of assignment of “ED case” status on the basis of clinical interview, preliminary analysis was conducted to confirm the validity of the “clinical” and “non-clinical” group identification, which was based on self-reported ED diagnosis. In Table 1, two groups were compared according to clinical impairment level, EDE-Q scores, and eating disorder behavior assessed by the EDE-Q. These include objective binge eating episodes (eating unusually large amount of food *and* having sense of losing control over eating), subjective binge eating episodes (having sense of losing control over eating *without* eating unusually large amount of food), and excessive exercising as a means of controlling weight or shape. Normative data for EDE-Q scores and prevalence of eating disorder behavior were also reported in Table 1 to ease comparison with present findings. As can be seen, participants with a self-reported ED had markedly elevated levels of ED symptoms when compared with the non-ED group, levels comparable to (but higher than) those of community cases of ED among young adult women identified by means of interview assessment in previous research.

### Comparison between measures

**Fig. 1:**
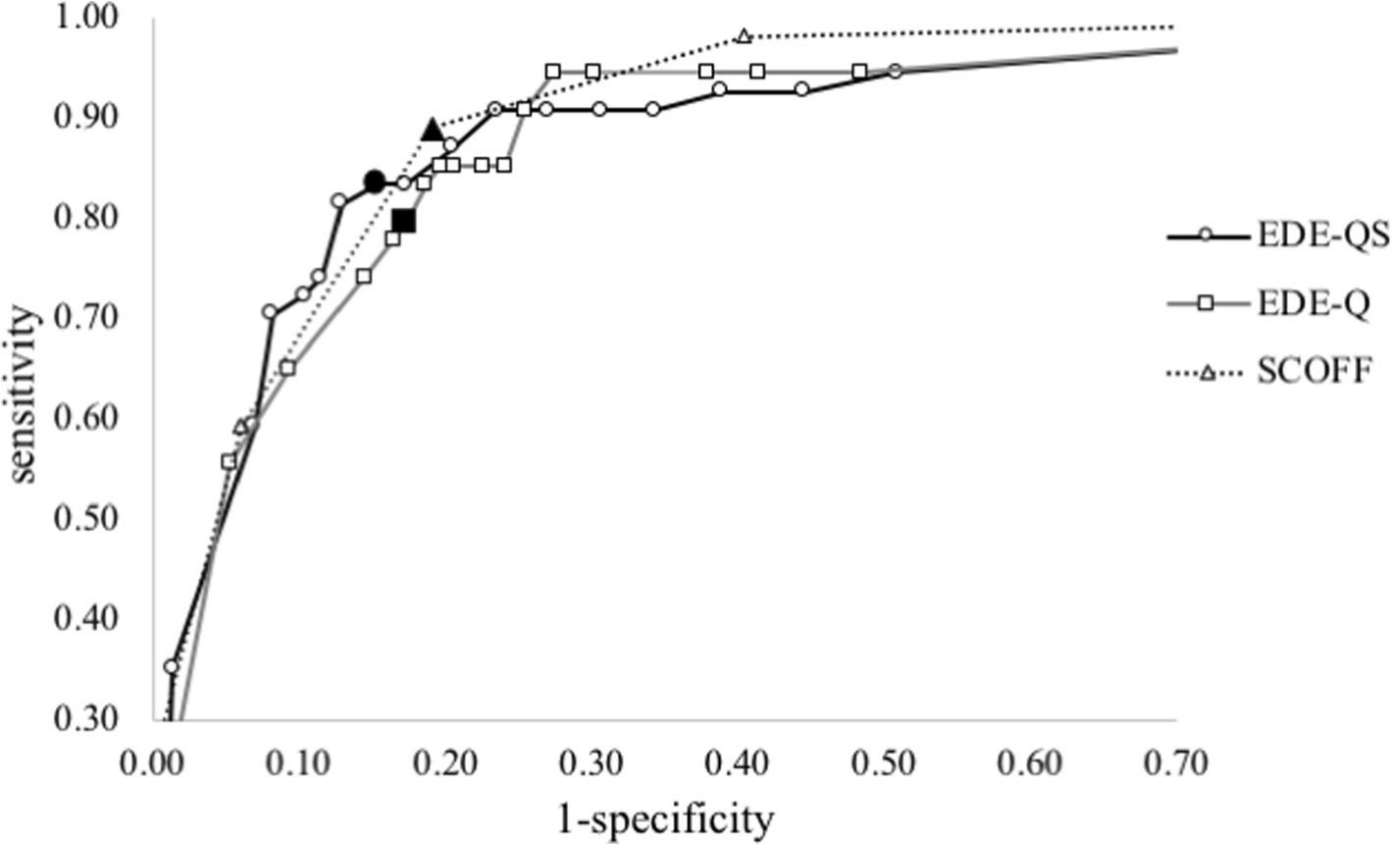
Relationship between sensitivity and specificity at different cut-off scores. Black-colored markers indicate cut-off scores of 15, 2.8, and 2 for EDE-QS, EDE-Q, and SCOFF, respectively

As shown in Fig. 1 and Table 2, the EDE-Q and EDE-QS demonstrated good, and the SCOFF excellent, discriminatory power according to the AUC statistic (AUC > .90) [18]. The EDE-Q showed an optimal sensitivity (.80) and specificity (.83) at a cut-off score of 2.8, where the PPV and NPV reached 33 and 97%, respectively. Similarly, with the cut-off score of ≥2, the SCOFF obtained a PPV and NPV of 34 and 99%, respectively. The EDE-QS demonstrated an optimal sensitivity (.83) and specificity (.85) at a total score cut-off of 15. At this cut-off, the NPV was very high (98%), as with the EDE-Q and SCOFF, while the PPV of 37% was higher than that for the EDE-Q (33%) or SCOFF (34%). Similar results were observed in the female subsample, with numerical differences in statistics of approximately 0.02 (higher sensitivity and PPV, lower specificity). Also, at a cut-off score of 15 on the EDE-QS, the proportion of probable ED cases in the total sample was 21.82%, which is somewhat lower than the rates yielded by the EDE-Q (23.26%) and SCOFF (25.58%), and closer to the proportion of participants’ self-reported ED diagnosis (9.66%).

**Table 2:** Summary statistics for various global score thresholds on the EDE-Q, EDE-QS (summation/average), and SCOFF

*Cut-off*	*Rate of probable ED (%)*	*Se(95%CI)*	*Sp(95%CI)*	*PPV(95%CI)*	*NPV(95%CI)*	*AUC(95%CI)*
EDE-Q						
2.6	25.94	.85(.73–.93)	.80(.77–.84)	.32(.24–.40)	.98(.96–.99)	88(.84–.93)
2.7	24.87	.83(.71–.92)	.81(.78–.85)	.32(.25–.41)	.98(.96–.99)	
2.8	23.26	.80(.66–.89)	.83(.79–.86)	.33(.25–.42)	.97(.95–.99)	
EDE-QS						
13/1.0	26.83	.87(.75–.95)	.80(.76–.83)	.31(.24–.39)	.98(.96–.99)	89(.84–.93)
14/1.1	23.61	.83(.71–.92)	.83(.79–.86)	.34(.26–.43)	.98(.96–.99)	
15/1.2	21.82	.83(.71–.92)	.85(.81–.88)	.37(.28–.46)	.98(.96–.99)	
16/1.3	19.50	.81(.69–.91)	.87(.84–.90)	.40(.31–.50)	.98(.96–.99)	
SCOFF						
2	25.58	.89(.77–.96)	.81(.77–.84)	.34(.26–.42)	.99(.97–.99)	.90(.87–.94)

*Note. ED* eating disorders, *Se* Sensitivity, *Sp* Specificity, *PPV* Positive Predictive Value, *NPV* Negative Predictive Value, *AUC* Area Under the Curve, *CI* Confidence Interval

### Comparison of alternative EDE-QS cut-off points

An EDE-QS score of 13 (but not 14 or 15) was within 1 SD of the mean score among participants who did not report an ED diagnosis, indicating that scores above 13 are likely to serve as cut-off points, if guided by suggestions in previous studies [20, 22]. Also, when utilizing the formula for detection of the criterion of clinical significance [23], defined as the mid-point between mean values of non-cases and cases, the result was 13.97. However, sensitivity was the same for cut-off points of 14 and 15, while specificity was greater with a cut-off point of 15. When comparing mean CIA total scores between self-reported ED cases and non-cases according to each of these proposed cut-off points, the difference was greatest with a cut-off point of 15 (*M*_diff_ = 20.05; 14: *M*_diff_ = 19.32; 13: *M*_diff_ = 18.53).

## Discussion

The EDE-QS demonstrated good discriminatory power, obtaining AUC, sensitivity, specificity and PPV estimates similar to those of the 28-item EDE-Q used in this study, and similar values were also reported in a study of young women in primary care [6]. Hence, reducing the number of items did not influence this instrument’s ability to differentiate between self-reported ED cases and non-cases. Similar discriminatory parameters where observed between the EDE-QS and the SCOFF as well, with a higher specificity but lower sensitivity produced by the EDE-QS. At the optimal cut-off point for the EDE-QS of 15, which yielded the best trade-off between sensitivity (.83) and specificity (.85) and the greatest divergence in clinical impairment between self-reported ED cases and non-cases, PPV was higher for the EDE-QS than for the SCOFF (.37 vs .34). A potential advantage of the EDE-QS is that it provides more information on specific ED behaviors and their severity, which could be more clinically useful and hence outweigh the cost of the additional time taken to complete few more items. For instance, the EDE-QS captures the frequency of binge eating and purging behavior, which is important for risk and health management.

It has been suggested [21] that when establishing cut-off points for ED screening tools, priority should be given to maximizing sensitivity (i.e. lowering the criteria for reaching ED level) rather than specificity, since the purpose of these instruments is to capture *potential* ED cases, the status of which could then be confirmed by means of further assessment, such as a clinical interview. Prioritizing sensitivity through the selection of a slightly lower cut-off point, while conducive to an over-inclusion of non-cases, would serve to ensure the inclusion of individuals experiencing or likely to experience clinically significant impairment associated with ED symptoms and could therefore be seen as good early intervention practice [24]. The current findings suggest that a cut-off point of 15 may be optimal when using the EDE-QS for screening purposes, although a slightly lower cut-off point may be preferable if optimizing sensitivity is the goal.

### Limitations and directions for future research

Some limitations should be noted. First, as in most previous studies of screening measures for ED, men were under-represented in the current study sample. Importantly, previous research has suggested that, when compared to thresholds established for women, a lower cut-off score on ED measures may be required to identify clinically significant ED symptoms in men [25, 26]. As the number of males with EDs in the current study was insufficient to conduct separate gender segregated analyses, further research will be needed to identify appropriate cut-off points for the EDE-QS (and other screening instruments) for men. Second, participants’ age ranged between 19 and 34, calling into question the representativeness of this group for the general population, including adolescents and elderly people. Relatedly, only 15% were classified as overweight or obese which might signify the shortfall of participants who experience regular binge eating episodes. Third, ED cases were self-reported rather than independently identified which is why inferences concerning direct application of the EDE-QS as a screening instrument at this stage are necessarily tentative. Further directions for future research hence include validation in general population and primary care samples using clinical interview as a reference point. Furthermore, while the fact that the EDE-QS captures ED symptoms over the past 7 days may be strength in terms of facilitating participant recall, the use of this relatively short time period - far less than the period of 3 months specified in formal diagnostic criteria - underscores the point that the EDE-QS, like the EDE-Q, is not intended as and should not be used as a diagnostic instrument. Lastly, to examine the suitability of the further use of the EDE-QS as a single-factor measure of ED psychopathology, additional directions for future research include employing parallel analysis for examination of the factor structure since this method has been shown to be superior to scree-plot analysis [27], that was used in the initial EDE-QS development study [4].

## Conclusion

In conclusion, as well as being a useful instrument for repeated assessments of people in treatment for an ED, the EDE-QS may be useful in screening for individuals likely to have clinically significant ED symptoms in non-clinical populations. In this study a cut-off score of 15 provided optimal validity coefficients.

## Data Availability

Original data is available as a supplementary file in the manuscript published by Gideon et al. (2016): 10.1371/journal.pone.0152744.s004

## Abbreviations

ED: Eating disorders
EDE-QS: Eating Disorder Examination Questionnaire Short
EDE-Q: Eating Disorder Examination Questionnaire
AN-R: Anorexia nervosa restrictive type
AN-BP: Anorexia nervosa binge eating/purging type
BN: Bulimia nervosa
BED: Binge eating disorder
OSFED: Other specified feeding and eating disorders
BMI: Body mass index
CIA: Clinical Impairment Assessment
ROC: Receiver operating characteristic
AUC: Area under the curve
PPV: Positive predictive value
NPV: Negative predictive value
